# Comparing adaptive tablet-based cognitive training and paper-and-pencil cognitive training: a pilot randomized controlled trial with community-dwelling stroke survivors

**DOI:** 10.1016/j.ijchp.2025.100627

**Published:** 2025-09-25

**Authors:** Joana Câmara, Sofia Carlos de Aguiar, Teresa Paulino, Ana Lúcia Faria, Sergi Bermúdez i Badia, Manuela Vilar, Eduardo Fermé

**Affiliations:** a Center for Research in Neuropsychology and Cognitive and Behavioral Intervention (CINEICC), Faculty of Psychology and Educational Sciences, University of Coimbra, Coimbra, Portugal; b NOVA Laboratory for Computer Science and Informatics, University of Madeira, Funchal, Portugal; c Agência para o Desenvolvimento da Investigação, Tecnologia e Inovação (ARDITI), Funchal; d Funchal Central Hospital (Dr. Nélio Mendonça, SESARAM), Funchal, Portugal; e Faculty of Exact Sciences and Engineering, University of Madeira, Funchal, Portugal; f Faculty of Arts and Humanities, University of Madeira, Funchal, Portugal; g Interactive Technologies Institute, LARSyS, Instituto Superior Técnico, Universidade de Lisboa, Lisbon, Portugal

**Keywords:** Tablet-based cognitive training, Paper-and-pencil cognitive training, Adaptive and personalized interventions, Stroke, Cognitive functioning, Emotional state, Quality of life, Functional abilities, Motivation

## Abstract

**Introduction:**

This study explored the feasibility and the preliminary efficacy of adaptive tablet-based cognitive training (CT) and paper-and-pencil CT approaches for mitigating multidomain post-stroke cognitive and noncognitive deficits.

**Methods:**

In this two-arm pilot randomized controlled trial, participants were randomly assigned to the NeuroAIreh@b (NAIr; adaptive tablet-based CT inspired by activities of daily living) and the Task Generator (TG; adaptive paper-and-pencil CT). A non-randomized passive control group was recruited for comparative purposes. Interventions comprised 12 bi-weekly 30-minute sessions. Primary outcomes explored training effects on several cognitive (e.g., global cognition, episodic memory), and noncognitive domains (e.g., quality of life, functional abilities).

**Results:**

A total of 20 participants were randomized (NAIr: *n* = 10; TG = 10). Within-group analysis revealed that the NAIr group presented significant improvements in more cognitive domains than the TG, and reported less functional disability, increased quality of life and greater motivation for rehabilitation at post-intervention. At follow-up, the NAIr group further improved in several cognitive domains and reported greater quality of life, while TG only improved in global cognition. Between-group analysis exhibited a pattern of superior performance in the adaptive CT groups over passive controls.

**Conclusions:**

Findings suggest that adaptive CT interventions are feasible to implement and lead to cognitive and noncognitive improvements in community-dwelling stroke survivors. However, while both training approaches yield different short and medium/long-term benefits, the NAIr – a more ecologically valid method – was the only to promote generalization of training effects to functionality and quality of life at post-intervention and three-month follow-up, respectively.

**Trial registration:**

The trial is registered at ClinicalTrials.gov, number NCT05929287. Registered July 3rd, 2023 (cf. https://classic.clinicaltrials.gov/ct2/show/NCT05929287).

## Introduction

Post-stroke cognitive impairment (PSCI) is highly prevalent and enduring among chronic stroke survivors, even after minor strokes, and is associated with increased dependency on activities of daily living (ADLs), higher likelihood of depressive symptoms and worse quality of life (Aam et al., 2020; Delavaran et al., 2017; Rohde et al., 2019). A large international study led by the Stroke and Cognition Consortium collected data from nine longitudinal hospital-based cohorts in seven countries (*n* = 1488), and concluded that, while survivors experience a substantial improvement in cognitive functioning in the first year after stroke, there is a significant decline in global cognition and all remaining cognitive domains (e.g., attention and processing speed, memory, language) over the next two years, with the exception of executive functions (Lo et al., 2022). Hence, the chronicity of PSCI has been estimated to anticipate the onset of dementia, with findings from the Oxford Vascular Study (OxVasc) suggesting that survivors of severe strokes present a dementia diagnosis 25 years earlier, compared with those with minor strokes (four years earlier) and transient ischemic attacks (two years earlier) (Pendlebury & Rothwell, 2019). Thus, given the negative long-term implications of PSCI research, investigating the efficacy of non-pharmacological interventions targeting PSCI in order to improve patients’ outcomes represents both a research and clinical priority (Lanctôt et al., 2020; Quinn et al., 2021).

Cognitive rehabilitation (CR) is an evidence-based personalized intervention that aims to improve cognitive, emotional and psychosocial outcomes in acquired brain injury patients, including stroke survivors (Fish & McKnight, 2021). The Cognitive Rehabilitation Task Force of the American Congress of Rehabilitation Medicine has issued 29 recommendations for evidence-based CR in several domains, including visual scanning for neglect after right-hemisphere stroke; compensatory strategies for mild memory deficits; language deficits after left-hemisphere stroke; social communication deficits after TBI; metacognitive strategy training for executive function deficits; and holistic neuropsychological rehabilitation to mitigate cognitive and functional disability after stroke or Traumatic Brain Injury (TBI) (Cicerone et al., 2019).

A key component of CR is cognitive training (CT), which consists of a restorative intervention technique with the aim of promoting cognitive functioning through the administration of repetitive, standardized and hierarchically organized tasks that are designed to address isolated cognitive functions (Clare & Woods, 2004; Clare et al., 2003; Pinto et al., 2023). The rationale behind CT is that routinely practicing these types of tasks can potentially improve or maintain the patient’s level of functioning in specific cognitive domains, with the expectation that these benefits may generalize beyond the training context (Clare & Woods, 2004; Clare et al., 2003). Traditional paper-and-pencil CT methods retain widespread adoption within rehabilitation settings due to their accessibility and established clinical validity (Faria et al., 2020; Parsons, 2016). However, a challenge of these methods lies in adapting training to patients, i.e., determining the appropriate intensity and difficulty levels according to patients’ needs. As Faria et al. (2018) noted, traditional paper-and-pencil CT delivery relies heavily on therapists’ clinical judgment. The absence of clear guidelines for paper-and-pencil CT – specifically in terms of administration and difficulty progression – introduces a certain level of subjectivity into the training process, making it challenging for therapists to hierarchically organize training tasks into a structured and adaptive CT program. Consequently, this places a heavy demand on therapists’, who are required to either create tasks from scratch or adapt existing tasks to meet patients’ rehabilitation needs, which then need to be manually corrected (i.e., scored) in order to determine the next set of training tasks based on patient performance. As a result, therapists are less likely to focus on the actual process by which patients perform tasks, neglecting to analyze specific aspects such as the difficulties faced when trying to solve the tasks; the type of errors committed; the type of strategies (effective or ineffective strategies) implemented to overcome some of the challenges posed by the training; the type of feedback (e.g., immediate, delayed) and the level of support required (e.g., physical assistance, modeling, general cues); the cues modalities (e.g., physical, verbal, visual); and the type of strategies that are more effective during task performance (e.g., internal compensation strategies, metacognitive strategies) (Sohlberg & Mateer, 2001; Sohlberg & Turkstra, 2011). The analysis of these processual aspects of CT is fundamental to enhance patients’ learning, provide suitable compensatory strategies to mitigate cognitive deficits and promote patients’ autonomy and independence in functionally oriented tasks (Sohlberg & Mateer, 2001; Sohlberg & Turkstra, 2011). Consequently, the challenges inherent in manually adapting CT can lead to the provision of suboptimal interventions (in terms of personalization and intensity), failing to adequately address the patient’s cognitive profile of strengths and weaknesses, thereby limiting the generalization of skills to everyday life (Faria & Bermúdez i Badia, 2015; Faria et al., 2018; Parsons, 2016).

The advancements in information and communication technologies (ICTs) have instigated the development of a myriad of computer-based CT programs (CCT) such as web-based CT platforms, telerehabilitation, and virtual reality (VR) systems, with the promises of assisting therapists in the provision of more personalized and cost-effective non-pharmacological interventions, and improving patients’ training experience and response to these interventions (Draaisma et al., 2020; Faria et al., 2024; Geraldo et al., 2018; Maggio et al., 2023; Riva et al., 2020). Indeed, these technologies integrate several features beneficial for the therapists, including the: i) high variability of training tasks, which are grouped according to target cognitive domains and subdomains; ii) instant and contingent feedback on the patient’s performance; iii) detailed progress tracking by resorting to various statistics and evolution graphs on patients’ performance (e.g., number of errors committed, overall performance, time needed to complete the task), and iv) automatic difficulty adjustment of CT tasks according to pre-defined criteria (Dores, 2012; Draaisma et al., 2020; Maggio et al., 2023; Oliveira et al., 2020). In addition, ICT-based CT technologies can promote patients’ adherence and engagement in the CT process due to immediate feedback contingent on each interaction, prompts and cue systems, and gamification factors (e.g., medals, score systems) (Faria et al., 2018; Oliveira et al., 2020; Nie et al., 2022).

Moreover, these technologies, particularly VR, offer the potential to increase the ecological validity of CT interventions (Câmara et al., 2021; Câmara, Ferreira et al., 2022; Faria et al., 2016; Riva et al., 2020). Indeed, VR environments can simulate real-world settings, such as supermarkets, shopping malls and cities, enabling the presentation of functionally oriented tasks embedded within these simulated environments (Faria et al., 2024; Parsons, 2016; Riva et al., 2020). However, establishing the ecological validity of neuropsychological interventions, including CT, requires a multifaceted approach beyond simply resorting to VR simulations of ADLs, involving, for instance, the assessment of patients’ goal attainment and functional abilities to analyze further if generalization of therapeutic gains has effectively occurred (Câmara et al., 2024; Pinto et al., 2024).

Evidence-based recommendations for clinical practice, such as those from the Cognitive Rehabilitation Task Force, strongly advocate for therapist involvement in ICT-based CT delivery, not supporting its stand-alone use for targeting cognitive deficits (Cicerone et al., 2019, 2022). These guidelines emphasize that ICT-based CT should be administered in conjunction with other therapeutic approaches directly supervised or monitored by a therapist. Indeed, therapist involvement in one-on-one sessions is crucial for promoting patients’ awareness, maintaining therapeutic gains and enhancing the generalization of training benefits to everyday life contexts, thus optimizing rehabilitation outcomes. However, the possibility of introducing remote training modalities to increase the intensity of CT interventions, beyond the scope of in-presence therapist-led sessions, must be carefully considered in light of patient characteristics. A systematic review conducted by Câmara et al. (2024) stated that acquired brain injury patients with moderate to severe physical, sensory and cognitive deficits are not suitable candidates for ICT-based CT self-led interventions, due to their increased need for feedback, monitoring, and support from the therapists and significant others. In contrast, the authors propose that self-led ICT-based CT interventions may be more appropriate for chronic acquired brain injury patients (specifically, stroke) exhibiting milder sequelae, especially when they have robust social support networks that can facilitate adherence and engagement throughout the rehabilitation process.

Research on the efficacy of ICT-based CT in comparison to traditional methods in stroke yields mixed results (Ho et al., 2021; Rogers et al., 2018). For instance, a systematic review and meta-analysis of six randomized controlled trials (RCTs) (*n* = 600) found no strong evidence for the superiority of CCT compared to traditional CT methods on stroke survivors’ overall cognitive function (Mingming et al., 2022). Similarly, another systematic review failed to support the existence of greater benefits arising from CCT compared to conventional interventions in global cognition and quality of life (Fava-Felix et al., 2022), as well as in working memory (Niemeijer et al., 2020). Conversely, one systematic review and meta-analysis of 17 RCTs (*n* = 622) demonstrated that CCT significantly improved global cognition, working memory, attention and executive function of stroke patients, but led to no significant improvements in ADLs and depressive symptomatology compared to control interventions (Zhou et al., 2022). These findings align with another systematic review of 28 studies (*n* = 768), further supporting the significant impact of CCT over traditional interventions for enhancing attention and executive functioning outcomes in both stroke and TBI patients (Bogdanova et al., 2015).

Furthermore, despite the ongoing debate on the efficacy of ICT-based CT compared to traditional CT in neurological patients, most research typically focuses on comparisons between adaptive ICT-based systems and non-adaptive traditional methods, i.e., CT methods lacking a standardized difficulty adaptation framework (Flak et al., 2019; Ho et al., 2022; Maier et al., 2020; Pedullà et al., 2016). This narrows our understanding of whether adaptive training approaches, regardless of their delivery method – computer-based versus paper-and-pencil – might positively impact stroke survivors' cognitive and noncognitive outcomes. In fact, to our knowledge the study conducted by Faria et al. (2020) appears to be the first to compare two content-equivalent adaptive CT methods to address PSCI: one delivered in desktop VR (Reh@City v2.0), in which patients navigated in a virtual city and performed a set of CT tasks incorporated within several ADLs (e.g., paying for the groceries in the supermarket, retrieving money from the ATM, cooking a meal in the kitchen), and another administered in paper-and-pencil (Task Generator [TG]), where patients were required to complete a set of traditional CT tasks with less ecologically valid stimuli (e.g., cancellation task with numbers, symbols and letters). The results of this one-month RCT with chronic stroke patients showed that both training approaches led to different immediate cognitive benefits: on one hand, the Reh@City v2.0 group exhibited significant improvements in measures of global cognitive functioning, attention, visuospatial ability and executive functions, while the TG group revealed improvements in measures of temporal-spatial orientation, processing speed, and verbal memory. Importantly, TG improvements were maintained at one-month follow-up, and new improvements were found in measures of sustained attention and language. However, Reh@City v2.0, which is considered to be a more ecologically valid CT approach, led to significantly greater improvements in general cognitive functioning, visuospatial abilities and executive functions in comparison to the TG. Despite these encouraging findings, the researchers did not analyze the impact of both training approaches on stroke survivors' subjective memory complaints, anxiety and depressive symptoms, quality of life, functional abilities, and motivation for rehabilitation. Notably, regarding the latter domain – functional abilities –, and considering that the Reh@City v2.0 presents VR-based simulations of ADLs, it would have been interesting to assess the generalization of the abovementioned cognitive improvements to survivors' ADLs.

Building upon the previous study by Faria et al. (2020), herein we propose a comparative study between two different adaptive CT methods, delivered in tablet and paper-and-pencil formats – the NeuroAIreh@b (NAIr) and the TG –, and a passive control group (waiting-list [WL]) not receiving any intervention. The NAIr platform represents a novel, adaptive tablet-based CT method developed through a participatory and multidisciplinary design approach involving stroke survivors, rehabilitation health professionals, informatics engineers and designers (Faria et al., 2024; Paulino, 2025). We believe that this platform provides a more ecologically valid training experience to patients through the inclusion of CT tasks inspired by instrumental activities of daily living (IADLs), which are embedded within real-world contexts, such as the kitchen, supermarket and the roadway. Additionally, the platform personalizes the CT demands by means of a two-step process: first, a comprehensive baseline neuropsychological assessment informs the initial set of parameters of the first five tablet-based CT tasks, and subsequently, supervised machine learning models continuously analyze patient performance data from each completed CT task to dynamically adapt the difficulty of the following tasks in order to ensure an optimal level of training challenge (Paulino, 2025). Thus, this pilot RCT aimed to assess the feasibility of two adaptive CT methods – NAIr and TG – and to compare their short and medium/long-term effects on a set of primary performance-based cognitive measures (e.g., global cognition, verbal memory, executive functions) and secondary self-report measures (e.g., motivation for rehabilitation, quality of life and functional abilities) in respect to a WL control group.

## Methods

This study was conducted between May 2023 and June 2024, and it was approved by the Madeira Health Service – SESARAM, EPERAM Ethical Committee (reference number 43/2019) and registered as a clinical trial on ClinicalTrials.gov (reference number: NCT05929287). The CONSORT 2015 checklist for reporting clinical trials is available in the Supplementary Data.

### Trial design

This is a two-arm RCT comparing both the NAIr and the TG interventions. In addition, a non-randomized WL group was recruited to serve as a control and facilitate further comparison of the effects of adaptive CT methods versus no intervention. All participants completed a neuropsychological assessment at three different stages – baseline (T0), post-intervention (T1) and three-month follow-up (T2). All neuropsychological assessments were conducted at two different sites: the Funchal Central Hospital (Dr. Nélio Mendonça, SESARAM) and the NeuroRehabLab research facilities. Parallel versions of the instruments were applied when available. The current trial underwent some necessary adjustments from the originally registered protocol, namely: (1) inclusion of the System Usability Scale (SUS) to assess the usability of the NAIr platform; and (2) recruitment of a non-randomized WL control group composed of the participants who, despite motivated to participate in the adaptive CT interventions, were not able to attend the required 12 sessions due to unavailability to comply with the training schedule, in result of geographic and transportation constraints. This study was not blind to the participant's allocation, as the psychologist responsible for conducting the assessments was the same involved in the intervention's delivery. Similarly, the non-randomized WL control group was not blinded to the study procedure; however, participants allocated to both experimental groups (EGs) were blinded to the specific intervention they were assigned (i.e., adaptive tablet-based CT or adaptive paper-and-pencil CT). Additionally, to minimize expectancy effects, they were not informed about the study’s primary objectives.

### Participants

**Fig. 1:**
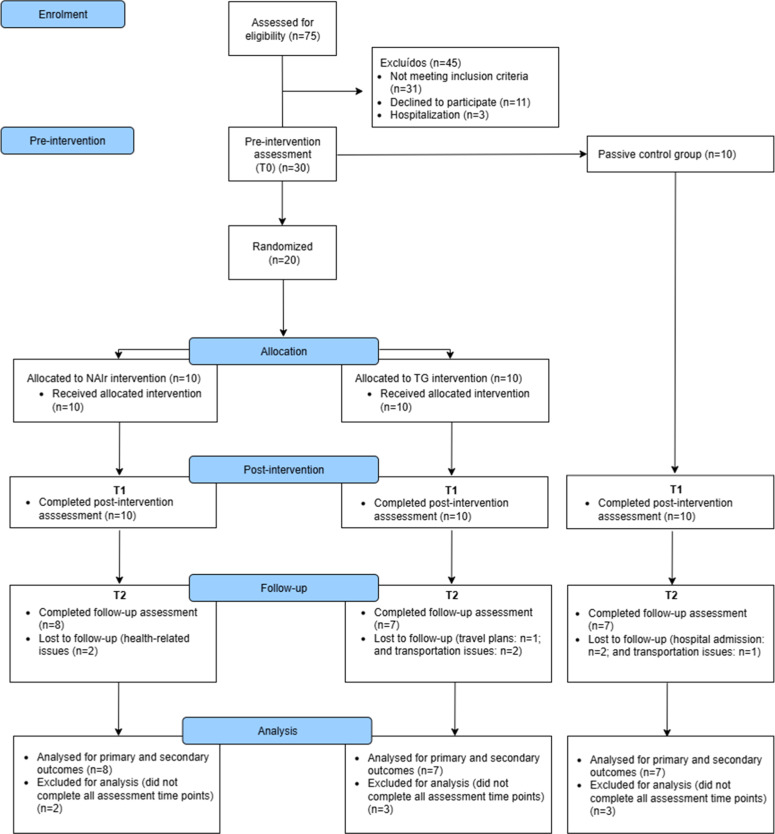
CONSORT 2025 diagram illustrating flow of participants through the study.

Participants were recruited at the Stroke Unit and the Physical Medicine and Rehabilitation Department (Funchal Central Hospital, SESARAM). The eligibility criteria were: (1) stroke diagnosis; (2) evidence of cognitive impairment on the Montreal Cognitive Assessment (MoCA) (≥ 1.5 standard deviations below the normative mean); (3) adults between 18 and 75 years old; (4) having at least three years of formal education; (5) relatively preserved language skills (expressive and receptive language); (6) residing in the community setting; (7) preserved visual and auditory acuity; (8) physically able to operate the tablet or to perform the paper-and-pencil training; and (9) motivation to participate. Participants were excluded if they had: (1) diagnosis of concomitant neurological and/or psychiatric disorders; (2) visual field defects (hemianopsia) and/or visual attention deficits (unilateral neglect); (3) and severe aphasia syndromes. A total of 75 participants were screened for eligibility. The study participant flow diagram is shown in Fig. 1.

### Interventions description

#### Adaptive tablet-based cognitive training: the NeuroAIreh@b platform

**Fig. 2:**
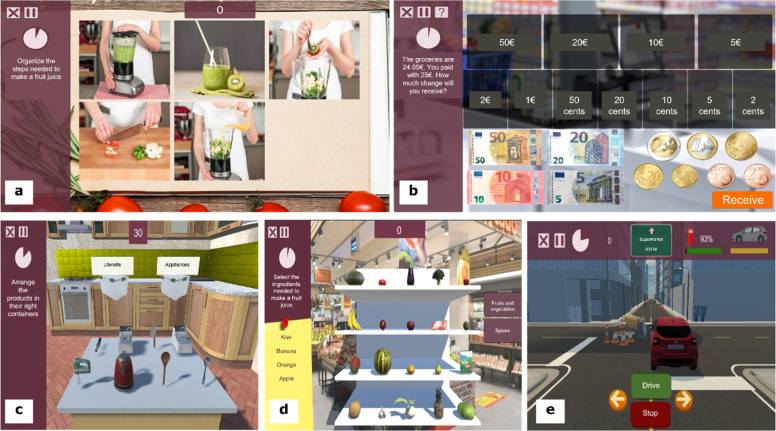
The NAIr CT tasks: (a) Reh@Org (action-sequencing); (b) Reh@Pay (calculation); (c) Reh@Cat (categorization); (d) Reh@Search (cancellation); and Reh@Drive (selective attention).

The NAIr is a tablet-based program in which participants are required to perform CT in five training applications (the Reh@Apps) with incorporated tasks of varying content and difficulty levels – the cancellation, action-sequencing, calculation, categorization and selective attention – within three everyday life scenarios (i.e., supermarket, the kitchen and the roadway (cf. Fig. 2). These CT tasks target attention, memory, language and executive functions.

The first set of five CT tasks is personalized based on each participant’s cognitive profile. This profile is established through a novel framework that aggregates participants’ raw scores on several objective neuropsychological measures. These are then integrated and organized into five main cognitive domains, specifically general cognition, attention, memory, language, and executive functions (see Faria et al., 2024, for a detailed explanation of this procedure).

**Fig. 3:**
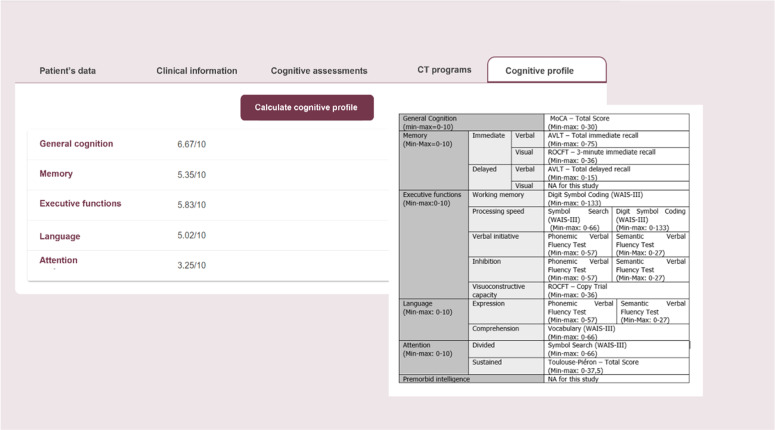
NexusBRaNT website: Baseline cognitive profile calculation process.

The psychologist utilizes the NexusBRaNT web platform to insert patients’ neuropsychological assessment data (Cunha, 2022). The NexusBRaNT serves as a comprehensive clinical management system, enabling therapists to 1) register patients eligible to participate in the tablet-based CT intervention; 2) input relevant clinical information, including the detailed neuropsychological assessment results; 3) prescribe adaptive training programs using the NAIr platform; and 4) monitor patients’ performance remotely if interventions are delivered at-distance. Following neuropsychological data insertion, the NexusBRaNT generates a five-domain cognitive profile for each participant, which is normalized on a scale ranging from 0 to 10 (cf. Fig. 3). Upon the profile generation, the Reh@Sync – a client-side interface for CT delivery within the NAIr – retrieves it from the database along with the training program settings (e.g., number of sessions, session duration, challenge range) and personalizes the first set of five CT tasks (Paulino, 2025). After each CT task iteration, performance data is sent to Reh@Sync and then compared with the pre-established challenge range (60–80 %). Difficulty adaptation is achieved through supervised machine learning models implemented within Reh@Sync. These models were trained using data collected from a prior pilot study (Câmara, Paulino et al., 2022; Paulino, 2025). Importantly, the Reh@Sync system continuously analyzes individual patient’s performance data across each task iteraction. Based on this analysis, the following adaptation guidelines are applied to modulate difficulty for subsequent tasks: a) participants performing below 60 % threshold are presented with a less difficult task; b) participants exceeding the 80 % threshold are presented with a more difficult task; and c) participants performing within 60–80 % range are presented with a task of similar difficulty (Paulino, 2025).

#### Adaptative paper-and-pencil cognitive training: the task generator

**Fig. 4:**
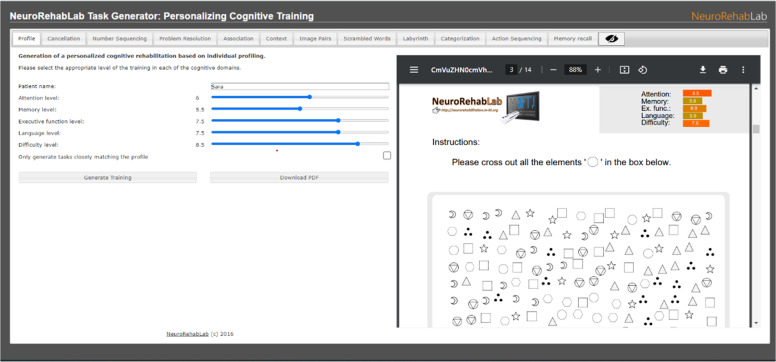
TG training personalization parameters (on the left), and the Cancelation task with symbols (on the right).

The TG is a free web-based CT tool that allows the generation of personalized paper-and-pencil CT sessions in a PDF format. Each PDF comprises 11 CT tasks, namely: Cancellation, Numeric Sequences, Problem Resolution, Association, Comprehension of Contexts, Image Pairs, Word Search, Mazes, Categorization, Action Sequencing, and Memory of Stories (cf. Fig. 4) (Faria et al., 2019, 2020).

First, the training was personalized according to the participant's cognitive profile, which was established through the baseline MoCA scores. The parameters available in the TG were defined as follows: (a) the Attention parameter was defined from the MoCA's Attention subdomain score (Min-Max: 0 – 6); (b) the Memory parameter by summing the scores obtained on the Delayed Recall and Orientation MoCA subdomain scores (Min-Max: 0 – 11); (c) the executive functions parameter by summing the Visuospatial, Executive and Abstraction MoCA subdomain scores (Min-Max: 0 – 7); (d) the Language parameter by summing the Naming and Language subdomain scores (Min-Max: 0 – 6), and (e) the Difficulty Parameter defined by the MoCA total score (Min-Max: 0 – 30) (Faria et al., 2019, 2020).

An Excel spreadsheet was created to insert participants' scores on the MoCA – subdomain scores and total scores – and to automatically normalize the raw scores on a scale ranging from 1 to 10. Consequently, it was possible to obtain the values of each TG parameter – Attention level, Memory level, Executive Function level, Language level, and Difficulty level, and insert them on the website to generate the initial set of tasks (Faria et al., 2019, 2020). Hence, participants' cognitive profile was represented by five main domains: attention, memory, language, executive function and global cognition.

Difficulty adaptation was performed after the participant completed the initial set of 11 CT tasks. Each CT task was scored (0–100 %) by a psychologist considering several criteria identified in the TG training manual.^1^ All task scores were then summed to obtain a mean performance score. The next session’s difficulty was determined by the following criteria: (a) on one hand, if the participants’ mean performance on the set of tasks ranged from 0–50 %, the difficulty parameter was reduced by 0.5; and (b), on the other hand, if the mean performance was higher than 70 %, the difficulty parameter was increased by 0.5 (Faria & Bermúdez i Badia, 2018; Faria al., 2018).

#### Waiting-list control group

Participants in the WL group were not performing any type of rehabilitation treatment at the time of the study and were instructed to continue their usual day-to-day routine. At the end of the study – after completing the three assessments planned throughout the study – they were offered the possibility of attending an adaptive CT intervention of their choice.

### Experimental setup

#### NeuroAIreh@b

**Fig. 5:**
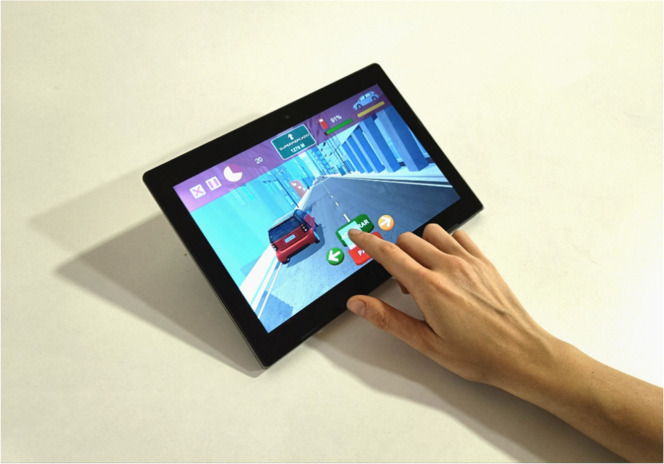
NAIr experimental setup. The user interacts with the training tasks through the touchscreen interface.

The NAIr was implemented using the Unity 3D game engine (Unity Technologies, San Francisco, USA). The experimental setup consisted of a tablet computer with a 10.1-inch display (Lenovo Ideapad D330 Tablet) running Windows 10 (CPU: Intel N4000 1.1 GHz, RAM: 4 G). For this study, the tablet’s keyboard was detached. Participants worked on a tabletop, and to improve ergonomics, the tablet display was positioned on a stand angled at 45 degrees (cf. Fig. 5).

#### Task generator

**Fig. 6:**
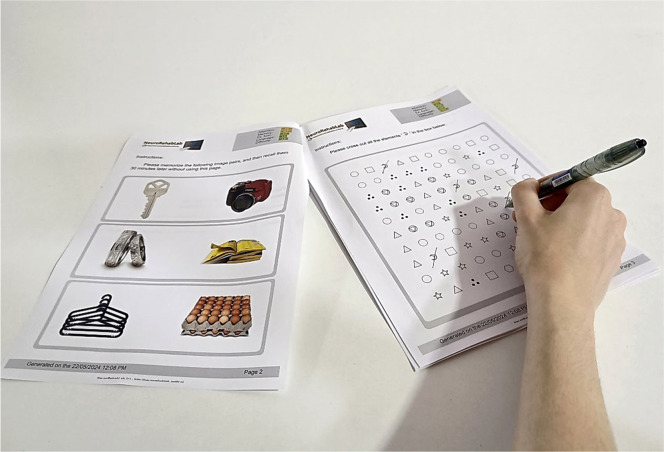
TG setup. The user completes the paper-and-pencil tasks with a pen or a pencil on a tabletop.

TG is an online application that does not require to be installed on the computer. The only required software was a PDF reader to open the downloaded paper-and-pencil CT tasks. After the tasks were printed, participants performed tasks on a tabletop using a pencil (cf. Fig. 6).

#### Outcome measures

We used the Portuguese versions of adapted and validated instruments for this study, with the exception of the Motivation for Traumatic Brain Injury Rehabilitation Questionnaire (MOT-Q), which is currently being validated within a master’s student’s research project for the Portuguese population with acquired brain injury. In this specific case, we relied on the Portuguese translation that was developed. Performance-based and self-report measures were completed at T0, T1 and T2, except for the Vocabulary Subtest of the Wechsler Adult Intelligence Scale – 3rd Edition (WAIS-III) (VOC; Wechsler, 2008), which was only administered at T0 to establish participants automated cognitive profile in the NAIr platform, a requirement for the personalization of the first set of cognitive training tasks.

#### Sociodemographic and clinical information questionnaire

Prior to the baseline neuropsychological assessment (T0), sociodemographic and clinical data were collected through a semi-structured interview during the eligibility assessment phase.

#### Feasibility outcomes

Feasibility was examined by considering two key indicators, namely the retention rates (i.e., the ratios between the number of participants that completed the post-intervention and the follow-up assessments, and the number of allocated participants for each experimental condition), and training adherence (i.e., compliance with the 12 training sessions).

### Primary outcome measures

#### Global cognition

Assessed by the Montreal Cognitive Assessment (MoCA; Freitas et al., 2011), a widely used cognitive screening tool that allows a brief assessment of different cognitive domains, such as attention, memory, language, visuoconstructional abilities, executive functions, mental calculation and orientation. The two Portuguese alternate forms of the MoCA were administered at T1 and T2 to prevent learning effects in serial assessments. The total score of the MoCA ranges from 0 to 30, with higher scores suggesting greater global cognition.

#### Processing speed

Assessed by the Digit Symbol-Coding (DS-C) and the Symbol Search (SS) subtests of the WAIS-III (Wechsler, 2008). In the DS-C, the participant is required to copy abstract symbols associated with numbers within a time limit of 120 s. The score ranges from 0 to 133. On the other hand, in the SS the participant is required to analyze a series of rows containing seven abstract symbols – two of them are the targets (on the right), and the remaining contain distractor symbols and one of the target symbols (on the left) –, and identify if one of the target symbols appears on the row on the left. If so, he/she should cross the option “Yes,” and if that is not the case, he/she should cross the option “No.” The task must be done within a time limit of 120 s, and the score ranges from 0 to 66. The DS-C and SS raw scores were converted to scaled scores (range: 1–19; Mean = 10, Standard deviation = 3). By summing the scaled scores of the DS-C and SS subtests, we obtained the processing speed index (PSI) (range: 50–150; Mean = 100, Standard deviation = 15). For this study, we will use the PSI index score when referring to participants' processing speed outcomes.

#### Sustained and selective attention

Assessed by the Toulouse-Piéron Cancellation Test (TP; Lima et al., 2021). The TP consists of a blank sheet of paper with 25 lines and 40 small squares per line. Each square contains a small line positioned in eight different directions. Participants are required to cross out all the squares that are identical to the three targets presented in the header for a total of 10 min. The assessment of participants’ performance requires the examiner to register the total number of hits (H) (targets correctly crossed out), errors (E) (false positives), and omissions (O) (false negatives) in order to be able to calculate three of the TP main outcomes: the Work Efficiency Index (WE), Dispersion Index (DI), and the Total Result (TR). The WE index is a measure of attentional and perceptual abilities and is calculated by the following formula: [WE = *H* – (*E* + *O*)]. The WE maximum score is 375; a higher score is indicative of greater attentional and perceptual abilities. On the other hand, the DI represents the percentage of mistakes the participant commits during the test and is calculated by the following formula: [DI = (*E* + *O*)/H x 100]. A higher score on the DI index is suggestive of a pattern of response characterized by distractibility and impulsivity. Finally, the TR is a measure of how much the participant effectively produces per minute and is calculated by the following formula: [TS = *H* (– *O* + *E* x 2 + 1)/10]. The TR ranges from −37.5 to 37.5, and the higher the TR, the better the participants’ performance. In this study, we have only considered participants' TR scores.

#### Verbal memory

Assessed by the Auditory Verbal Learning Test (AVLT; Cavaco et al., 2015), a 15-item word list verbal memory test. The examiner reads a list of 15 unrelated high-frequency words, and the participant is asked to recall as many words as possible spontaneously. In total, there are five learning trials. Thirty minutes later, the participant is presented with a delayed recall trial, in which he/she must spontaneously recall as many words as possible. Finally, the task is completed with a delayed recognition trial, where the examiner reads a 30-item word list, and the participant is required to identify by means of a “Yes” or “No” answer, the words that belonged to the 15-item world list read by the examiner five times, and the words that were not a part of that list. A higher score on all six trials – immediate recall trials from 1 to 5 and delayed recall trials – reflects a better performance. For this study, we used two scores: the AVLT total immediate recall score (AVLT 1 – AVLT 5; AVLT-TIR) and the AVLT delayed recall score (AVLT-DR).

#### Visual memory

Assessed by the Rey-Osterrieth Figure Complex Test – 3-minute immediate recall trial (ROCFT; Espírito-Santo et al., 2015). The ROCFT is a neuropsychological instrument that assesses a broad spectrum of cognitive processes. However, this measure has been mostly associated with the assessment of visuospatial/constructional abilities, visual memory, and executive functioning abilities, specifically problem-solving, organization and planning (Sherman et al., 2023). The three-minute immediate recall trial targets short-term visual memory, and the participant is required to draw the figure that was previously copied from memory. The figure is composed of a total of 18 elements, and each element is scored on a 0 (absent or not recognizable) to 2 (correctly reproduced and placed properly). The total score ranges from 0 to 36, with higher scores reflecting a better performance on the task.

#### Language

Assessed by the Vocabulary subtest of the WAIS-III (VOC; Wechsler, 2008). This subtest requires the participant to provide oral definitions for 33 progressively more complex words, varying from concrete to abstract concepts. The subtest is discontinued after six consecutive failures. Items are scored on a 0 to 2 scale based on specific criteria that are detailed in the manual. Raw scores ranged from 0 to 66 and were used to compute participants' cognitive profiles in the NAIr platform. For this study, we do not analyze participants' performance on the VOC subtest as this measure was only administered at T0.

#### Visuoconstructional abilities and executive functions

Assessed by the copy trial of the ROCFT (Espírito-Santo et al., 2015) and the Verbal Fluency Tests (VFT; Cavaco et al., 2013), more specifically through the phonemic verbal fluency (PVF) (letters R, M and P) and the semantic fluency (SVF) (animals' category) tests. Regarding the copy trial of the ROCFT, the examiner places a figure in front of the participant, and he/she is required to copy it. The total score ranges from 0 to 36, and the higher the score, the better the participant's performance. In the SVF test, the participant is asked to generate the names of as many species of animals as possible within one minute. Moreover, in the PVF test, the participant is required to produce orally as many words as possible beginning with a specific letter (R, M and P) within one minute. The total score of the SVF test corresponds to the number of animal species named within the specified time frame, and the total score of the PVF test consists of the sum of the three trials (letters R, M and P). In both tests, the higher the scores, the better the performance.

### Secondary outcome measures

#### Subjective memory complaints

Measured by the Subjective Memory Complaints Scale (SMCS; Ginó et al., 2015), a 10-item self-report rating scale comprising several daily life memory-related activities. The participant is required to rate each item according to different rating scales, reflecting the absence and presence of subjective memory complaints (e.g., in the item “Do you have memory complaints?”, the rating scale is composed of the options: no; yes, but it has little relevance; yes, and they are very relevant, yes, it is very troublesome). The total score of the SMCS ranges from 0 (absence of subjective memory complaints) to 21 (maximum level of subjective memory complaints), with a cut-off score of ≥ 3 indicating the presence of significant subjective memory complaints.

#### Anxiety and depressive symptoms

Measured by the Hospital Anxiety and Depression Scale (HADS; Pais-Ribeiro et al., 2007), a screening questionnaire consisting of two subscales – anxiety and depression – with seven items each. Each item is scored on a 4-point Likert scale, ranging from 0 to 3, and both subscales maximum score ranges from 0 to 21. For this study, we considered the HADS total score and the cut-off of 11 points for establishing the presence of clinically meaningful anxiety and depressive symptoms.

#### Quality of life

Assessed by the Quality of Life after Brain Injury Questionnaire (QOLIBRI; Guerreiro et al., 2014), a comprehensive 37-item self-report questionnaire covering six dimensions of health-related quality of life: Cognition, Self, Daily life and Autonomy, Social Relationships, Emotions and Physical problems. The first four scales, i.e., Cognition, Self, Daily Life and Autonomy, and Social Relationship scales, assess participants’ satisfaction with these dimensions of life, and items are coded on a 1 to 5 scale, where 1 means “not satisfied at all” and 5 “very satisfied.” The two final scales, i.e., Emotions and Physical Problems, assess in what way participants are bothered by their emotional and physical issues, and items are coded on a 1 to 5 scale, where 1 means “very bothered” and 5 means “not at all bothered.” From the QOLIBRI, it is possible to obtain 7 different scores – the 6 subscales scores plus a total score. The QOLIBRI scores are reported on a 0-100% scale, where 0 is indicative of the worst possible quality of life and 100 is indicative of the best possible quality of life. For this study, we used the QOLIBRI total score.

#### Functional abilities

Assessed by the Adults and Older Adults Functional Assessment Inventory (IAFAI; Sousa et al., 2015). The IAFAI is a 50-item comprehensive questionnaire used to measure the self-perceived functional incapacity of adults and older adults. The IAFAI encompasses three types of activities of daily living: Basic Activities of Daily Living (BADL) (Feeding, Depressing, Bathing and Continence, and Mobility and Transference); Instrumental Activities of Daily Living – Household (IADL-H) (Conversation and Telephone Use, Meal Preparation, Housekeeping, and Home Security); and Instrumental Activities of Daily Living – Advances (IADL-A) (Comprehensive and Communication, Health-related Decision Making, Finances, Going Out and Transportation Use, and Leisure and Interpersonal Relationships).

For each daily living activity, there are four possible outcomes: independence, independence with difficulty, dependence or non-applicable. Additionally, for each item where functional incapacity is identified (i.e., independence with difficulty and dependence), it is possible to select the main reason responsible for the incapacity, i.e., if it is caused mainly by physical, cognitive or emotional factors. It is possible to obtain seven different scores: global functional incapacity, BADL incapacity, IADL-H incapacity, IADL-A incapacity, physical incapacity, cognitive incapacity, and emotional incapacity. Scores range from 0 to 100 %, and higher scores indicate worse functional status. For this study, we have considered the global functional incapacity score.

#### Motivation for rehabilitation

The (MOT-Q) (Chervinsky et al., 1998) was used to assess participants' motivation and engagement in rehabilitation. Although the MOT-Q was validated on patients with TBI, it has been found to have good face validity for acquired brain injury patients in general. We have used the adapted Portuguese version of the questionnaire, which is currently undergoing validation for Portuguese acquired brain injury patients. The MOT-Q is a self-report 31-item scale in which items are organized into four subscales: interest in rehabilitation, reliance on professional help, lack of denial, and lack of anger. The total possible score on this self-report scale is 62, and higher scores reflect higher engagement in rehabilitation.

#### Usability

Finally, following the NAIr intervention, the SUS (Brooke, 1996) was used to assess patients’ satisfaction and usability with the NAIr. The SUS score ranges from 0 to 100, where higher scores are suggestive of higher usability levels. The interpretation of the SUS scores was performed according to the criteria proposed by Sauro and Lewis (2012).

#### Procedures

After giving written informed consent, eligible participants completed the baseline neuropsychological assessment at the hospital or in the NeuroRehabLab research institution facilities and were randomized into one of two groups: NAIr or TG. Concerning the latter, WL participants were offered a CT intervention of their choice following the completion of the research project. Participants’ randomization to the experimental groups (EGs) was carried out by an independent researcher through a simple randomization method using a website that generates a random allocation sequence (cf. http://www.randomization.com). Then, a certified psychologist and doctoral student in neuropsychology (JC) delivered the individual, 12 biweekly 30-minute sessions of adaptive CT interventions to the stroke survivors. All CT sessions were conducted in a supervised setting, either at Funchal Central Hospital (Hospital Dr. Nélio Mendonça, SESARAM) or at the NeuroRehabLab research facilities, where they were closely monitored and supervised by the psychologist to ensure protocol adherence and provide support as needed. Finally, participants underwent reassessments at one week and three months following the intervention by the same psychologist who delivered the intervention. Neuropsychological assessments were conducted at the aforementioned sites.

#### Statistical analysis

Statistical analyses were performed using the SPSS software (version 26, SPSS, Inc., Chicago IL, USA). Normally distributed continuous variables were presented as mean and standard deviation, non-normally distributed variables as medians and interquartile ranges (IQR), and categorical variables as frequencies. Group differences in demographic variables, stroke-related variables, and baseline level of functioning in the various primary and secondary outcome measures were analyzed using the Kruskal-Wallis test and the Fisher Exact Test. As most data were not normally distributed, non-parametric tests were employed. The Wilcoxon signed-rank test was performed to evaluate participants' within-group differences from baseline to post-intervention and baseline to follow-up. Then, the Kruskal-Wallis test was used to assess participants’ between-group differences from baseline to post-intervention (i.e., immediate effects) and baseline to follow-up (i.e., medium/long-term effects). When statistically significant differences were detected (effect of group), post hoc comparisons were performed between the groups using the Mann-Whitney U tests. Effect size (*r*) estimates were calculated (*r* = *Z*/√N) and interpreted as 0.2 = small, 0.5 = medium, and 0.8 = large (Cohen, 1988). No corrections for multiple comparisons were performed. In all analyses, a significance level of α = 0.05 was applied.

## Results

### Sample description

The sample consisted of 20 stroke survivors randomly distributed in two EGs – NAIr (EG I) and TG (EG II). The EG I (NAIr) comprised 10 participants with stroke (3 male, 7 female) with an average of 54.50 (SD = 9.93) years of age, 12.49 (SD = 5.54) formal years of education, and 19.70 (SD = 25.45) months post-stroke. The TG group was composed of 10 participants (8 male, 2 female), with an average of 57.30 (SD = 13.55) years of age, 10.20 (SD = 5.87) formal years of education, and 23.50 (SD = 19.25) months post-stroke. Finally, in the WL group, there were 10 participants (4 male, 6 female), with an average of 62.20 (SD = 10.47) years of age, 10.40 (SD = 4.06) formal years of education, and 15.20 (SD = 12.54) months post-stroke. The Kruskall-Wallis test revealed no differences between groups in age, education, time post-stroke, and baseline neuropsychological outcome measures (all *p values* ≥ 0.05). In addition, there were also no differences between groups in terms of the stroke subtype and stroke location according to the Fisher Exact Test (all *p values* ≥ 0.05) (cf. Table 1).

**Table 1:** Participants sociodemographic and stroke-related characteristics, as well as baseline level of functioning in the different neuropsychological outcome measures.

	NAIr (*n* = 10)	TG (*n* = 10)	WL (*n* = 10)	*p value*
Age, years [(Mean, SD)]	54.50 (9.93)	57.30 (13.55)	62.20 (10.47)	.237^a^
Gender, n (F/M)	7/3	2/8	6/4	.879^b^
Education, years [(Mean, SD)]	12.40 (5.54)	10.20 (5.87)	10.40 (4.06)	.591^a^
Stroke subtype (I/H)	4/6	5/5	8/2	.171^b^
Stroke location (R/L/NS)	6/4	3/7	2/7/1	.452^b^
Time post-stroke, months [(Mean, SD)]	19.70 (25.45)	23.50 (19.25)	15.20 (12.54)	.624^a^
MoCA [Mdn (IQR)]	19.50 (6.5)	20.00 (7.25)	21 (3.25)	.695^a^
PSI [Mdn (IQR)]	99 (37.5)	90 (17.75)	104.5 (19.5)	.149^a^
TP, TR [Mdn (IQR)]	9.65 (9.4)	7.4 (9.78)	9.85 (9.35)	.779^a^
SVF, animals [Mdn (IQR)]	14.5 (7.75)	12 (4)	15.50 (7.75)	.537^a^
PVF, PRM letters [Mdn (IQR)]	22 (15.5)	17.50 (18.5)	18.50 (13.5)	.661^a^
AVLT, TIR [Mdn (IQR)]	28.50 (14)	37.50 (14.75)	29.50 (16)	.499^a^
AVLT, DR^30 min^ [Mdn (IQR)]	5.5 (4)	6.50 (7.75)	5.00 (3.75)	.673^a^
ROCFT, Copy [Mdn (IQR)]	31.25 (12)	26.5 (19)	28.00 (14.12)	.731^a^
ROCFT, IR^3 min^ [Mdn (IQR)]	6.75 (12.87)	8.5 (19)	9.25 (11)	.815^a^
SMCS [Mdn (IQR)]	5.50 (8.25)	7.50 (9)	6.00 (4.5)	.485^a^
HADS, total [Mdn (IQR)]	11.50 (9.75)	14.00 (14.75)	14.50 (11)	.721^a^
QOLIBRI, total [Mdn (IQR)]	56.42 (22.85)	51.69 (31.93)	55.74 (13.61)	.649^a^
IAFAI, total [Mdn (IQR)]	25.36 (23.74)	42.36 (29.8)	31.73 (28.93)	.068^a^
MOT-Q [Mdn (IQR)]	39 (13.25)	42.50 (18.75)	30.50 (19)	.083^a^

Note: AVLT = Auditory Verbal Learning Test; DR^a^ = Delayed Recall (30-min); *F* = Female; *H* = Hemorrhagic; HADS = Hospital Anxiety and Depression Scale; *L* = Left; *I* = Ischemic; IAFAI = Adults and Older Adults Functional Assessment Scale; IR^b^ = Immediate Recall (3-min); *M* = Male; MoCA = Montreal Cognitive Assessment; MOT-*Q* = Motivation for Traumatic Brain Injury Rehabilitation QuestionNAIre (MOT-Q); NS = Not specified; PSI = Processing Speed Index; QOLIBRI = Quality of Life after Brain Injury; *R* = Right; ROCFT = Rey-Osterrieth Complex Figure Test; TIR = Total Immediate Recall; TP = Toulouse-Piéron; TR = Total Result; SMCS = Subjective Memory Complaints Scale; SS = Symbol Search; SVF = Semantic Verbal Fluency.

a Kruskal-Wallis.

b Fisher Exact Test.

### Feasibility outcomes

In terms of the interventions’ feasibility, both the NAIr and TG groups demonstrated high levels of retention and training adherence. The post-intervention completion rates were 100 % for both groups. Also, participants from both groups attended all scheduled 12 CT sessions, revealing a 100 % attendance rate and a high level of engagement throughout the intervention duration. At the three-month follow-up, the completion rate was 80 % (*n* = 8) in the NAIr group and 70 % (*n* = 7) in the TG group. Two participants in the NAIr group were lost to follow-up due to unforeseen reasons, which were not related to their engagement and motivation for the intervention, specifically health-related issues. In addition, three participants in the TG group were lost to follow-up for other reasons than adverse responses to the intervention, namely travel plans (*n* = 1) and difficulties in attending the session due to transportation issues (*n* = 2).

### Primary outcome measures

The primary outcome measures scores from the three groups in the different assessment moments are described in Table 2. A within-group analysis revealed that the NAIr group exhibited significant post-intervention improvements in the MoCA (*W*_(10)_ = 26, *Z* = −2.047, *p* = .041, *r* = 0.65), TP-TR (*W*_(10)_ = 48; *Z* = −2.091; *p* = .037, *r* = 0.66), PVF (*W*_(10)_ = 36, *Z* = −2.527; *p* = .012, *r* = 0.80), and in the ROCFT-C (*W*_(10)_ = 26.5, *Z* = −2.120, *p* = .034, *r* = 0.67). In contrast, the TG group revealed significant post-intervention improvements in the TP-TR (*W*_(10)_ = 50, *Z* = −2.293; *p* = .022, *r* = 0.73), and the PVF (*W*_(10)_ = 47, *Z* = −1.992; *p* = .046, *r* = 0.63). In addition, the WL group presented significant post-intervention declines in the SVF (*W*_(10)_ = 5.5, *Z* = −2.023; *p* = .43 , *r* = 6.40), and the AVLT-DR (*W*_(10)_ = 4, *Z* = −2.221; *p* = .026, *r* = 0.070).

**Table 2:** Primary outcomes comparisons at pre, post-intervention and follow-up.

Measures	NAIr			TG			WL		
	**Pre (*n* = 10) Mdn (IQR)**	**Post (*n* = 10) Mdn (IQR)**	**FU (*n* = 8) Mdn (IQR)**	**Pre (*n* = 10) Mdn (IQR)**	**Post (*n* = 10) Mdn (IQR)**	**FU (*n* = 7) Mdn (IQR)**	**Pre (*n* = 10) Mdn (IQR)**	**Post (*n* = 10) Mdn (IQR)**	**FU (*n* = 7) Mdn (IQR)**
MoCA	19.50 (6.5)	22.00 (2.25)	23.50 (4.5)	20.00 (7.25)	21.50 (6.5)	22 (7.25)	21 (3.25)	20.5 (4.75)	19 (3)
*p* value*^w^*_(T0-T1)_	.041*			.101			.064		
*p* value*^w^*_(T0-T2)_	.042*			.048*			.027*		
PSI	99 (37.5)	98 (37.5)	111 (51.5)	90 (17.75)	90 (17.75)	90 (29.25)	104.5 (19.5)	100 (12.75)	98 (29)
*p* value*^W^*_(T0-T1)_	.085			.524			.079		
*p* value*^W^*_(T0-T2)_	.197			.197			.027*		
TP, TR	9.65 (9.4)	10.85 (9.2)	13.10 (13.45)	7.4 (9.78)	8.9 (14.63)	8.4 (11.2)	9.85 (9.35)	6.95 (8.67)	6.4 (6.4)
*p* value*^W^*_(T0-T1)_	.037*			.022*			.139		
*p* value*^W^*_(T0-T2)_	.012*			.735			.034*		
SVF, animals	14.5 (7.75)	13.5 (7.75)	15.5 (3)	12 (4)	13.5 (7.75)	14 (7)	15.5 (7.75)	12 (6.5)	11 (8)
*p* value*^W^*_(T0-T1)_	.918			.437			.043*		
*p* value*^W^*_(T0-T2)_	.121			1.00			.084		
PVF, PRM letters	22 (15.5)	28 (14)	27.5 (10.5)	17.5 (18.5)	21 (13.25)	25 (15)	18.5 (13.5)	16.5 (14.25)	13 (9)
*p* value*^W^*_(T0-T1)_	.012*			.046*			.123		
*p* value*^W^*_(T0-T2)_	.270			.176			.057		
AVLT, TIR	28.5 (14)	34 (6)	40.50 (13.25)	37.50 (14.75)	40.5 (17.25)	36 (15)	29.50 (16)	25.5 (15.75)	20 (6)
*p* value*^W^*_(T0-T1)_	.114			.108			.074		
*p* value*^W^*_(T0-T2)_	.050*			.686			.018*		
AVLT, DR	5.5 (4)	5.5 (5.75)	7.5 (1.75)	6.5 (7.75)	7.5 (5.25)	8 (6)	5 (3.75)	4.5 (3.75)	3 (5)
*p* value*^W^*_(T0-T1)_	.170			.521			.026*		
*P* value*^W^*_(T0-T2)_	.017*			.854			.078		
ROCFT, Copy	31.25 (12)	34 (8.25)	35 (15.87)	26.5 (19)	28.25 (6)	26.5 (24)	28 (14.12)	23.5 (13.5)	21 (17)
*p* value*^W^*_(T0-T1)_	.034*			.176			.406		
*p* value*^W^*_(T0-T2)_	.051			.588			.027*		
ROCFT, IR	6.75 (12.88)	10.5 (8.50)	14.50 (11.76)	8.5 (19)	12.25 (9.5)	15 (18.5)	9.25 (11)	10.50 (13.12)	7 (7)
*p* value*^W^*_(T0-T1)_	.139			.213			.799		
*p* value*^W^*_(T0-T2)_	.172			.345			.104		

Note: AVLT = Auditory Verbal Learning Test; DR^a^ = Delayed Recall (30-min); *F* = Female; *H* = Hemorrhagic; *L* = Left; *I* = Ischemic; IR^b^ = Immediate Recall (3-min); *M* = Male; MoCA = Montreal Cognitive Assessment; NS = Not specified; PVF = Phonemic Verbal Fluency; PSI = Processing Speed Index; *R* = Right; ROCFT = Rey-Osterrieth Complex Figure Test; TIR = Total Immediate Recall; TP = Toulouse-Piéron; TR = Total Result; SVF = Semantic Verbal Fluency; *p* value*^W^* = within-group changes (by time of assessment): **p < .05; **p < .01.

At post-intervention, a Kruskal-Wallis test revealed that participants’ performance on several primary outcome measures was significantly affected by the type of treatment condition (cf. Table 3). Specifically, there were significant between-group differences in the MoCA (*χ2*_(2)_ = 8.677, *p* = .013), PSI (*χ2*_(2)_ = 9.411, *p* = .009), TP-TR (*χ2*_(2)_ = 9.123, *p* = .010), PVF (*χ2*_(2)_ = 9.417, *p* = .009), AVLT-TIR (*χ2*_(2)_ = 8.478, *p* = .014), and the AVLT-DR (*χ2*_(2)_ = 6.406, *p* = .041). Post hoc comparisons revealed that participants in both interventions groups demonstrated a significantly better performance than participants in the WL group in the MoCA (TG vs. WL: *U* = 9.1, *Z* = −2.344, *p* = .019, *r* = 0.74; NAIr vs. WL: *U* = 10.55, *Z* = −2.717, *p* = .007, *r* = 0.86), TP-TR (TG vs. WL: *U* = 9.4, *Z* = 2.389, *p* = .017, *r* = 0.90; NAIr vs. WL: *U* = 11, *Z* = 2.795, *p* = .005, *r* = 0.988), PVF (TG vs. WL: *U* = 10.15, *Z* = −2.582, *p* = .10, *r* = 0.817; NAIr vs. WL: *U* = 10.700, *Z* = −2.725, *p* = .006, *r* = 0.86), AVLT-TIR (TG vs. WL: *U* = 8.8, *Z* = −2.244, *p* = .025, *r* = 0.71; NAIr vs. WL: *U* = 10.7, *Z* = −2.729, *p* = .006, *r* = 0.86), and AVLT-DR (TG vs. WL: *U* = 7.6, *Z* = −1.957, *p* = .050, *r* = 0.62; NAIr vs. WL: *U* = 9.2, *Z* = −2.369, *p* = .018, *r* = 0.75). In addition, participants in the WL group exhibited a significant decline on the PSI when compared to participants in the NAIr group (NAIr vs. TG: *U* = 11.95, *Z* = −3.061, *p* = .002, *r* = 0.97).

**Table 3:** Post hoc comparisons across groups considering the primary outcome measures.

Measures	NAIr	TG	WL	KW (T1-T0)	MW NAIr vs. TG	MW NAIr vs. WL	MW TG vs. WL	NAIr	TG	WL	KW (T2-T0)	MW NAIr vs. TG	MW NAIr vs. WL	MW TG vs. WL
	**Mdn T1-T0 (IQR)**	**Mdn T1-T0 (IQR)**	**Mdn T1-T0 (IQR)**	***χ2 (p value)***	***U* (*p* value)**	***U* (*p*-value)**	***U* (*p* value)**	**Mdn T2-T0 (IQR)**	**Mdn T2-T0 (IQR)**	**Mdn T2-T0 (IQR)**	***χ2 (p value)***	***U* (*p* value)**	***U* (*p* value)**	***U* (*p* value)**
MoCA	1 (3.25)	1 (3)	−1.5 (3.25)	8.677* (0.013)	1.45 (0.709)	**10.55^‡^ (0.007)**	**9.1^†^ (0.019)**	3 (4.5)	1 (2)	−2 (4)	11.076* (0.004)	1.527 (0.648)	**10.455‡ (0.002)**	**8.929^†^ (0.010)**
PSI	6.5 (13.75)	0 (4.25)	−3 (8.5)	9.411* (0.009)	6.65 (0.088)	**11.95^‡^ (0.002)**	5.3 (0.175)	8.5 (12.5)	1 (6.25)	.8 (23)	9.659* (0.008)	1.333 (0.688)	**9.357‡ (0.003)**	**8.024^†^ (0.019)**
TP, TR	3.05 (5.03)	1.55 (2.17)	−1.5 (3.28)	9.123* (0.010)	1.6 (0.407)	**11^‡^ (0.005)**	**9.4^†^ (0.017)**	2.25 (3.6)	.7 (3.7)	−2.6 (2.7)	11.235* (0.004)	5.045 (0.133)	**11.259‡ (0.001)**	6.214 (0.073)
SVF, Animals	−0.5 (10.25)	2 (6.75)	−2 (5)	3.319 (0.190)	43.5 (0.621)	37 (0.353)	24.5 (0.052)	1 (3)	0 (7)	−1 (3.25)	4.437 (0.109)	22.5 (0.536)	10‡ (0.021)	21 (0.412)
PVF, PRM letters	3.5 (4.25)	3 (6)	−3 (4.5)	9.417* (0.009)	.550 (0.889)	**10.7^‡^ (0.006)**	**10.15^†^ (0.010)**	5 (9.25)	5 (11)	−2 (9)	5.492 (0.064)	26.5 (0.861)	11.5‡ (0.031)	12.5 (0.070)
AVLT, TIR	7 (10)	4.5 (9)	−4.5 (3.25)	8.478* (0.014)	1.9 (0.628)	**10.7^‡^ (0.006)**	**8.8^†^ (0.025)**	6 (8.5)	0 (7)	−8 (14)	14.080* (0.001)	5.875 (0.080)	**12.589 (<0.001)**	6.714 (0.053)
AVLT, DR	1 (4.25)	0 (3)	−2 (2.5)	6.406* (0.041)	1.6 (0.680)	**9.2^‡^ (0.018)**	**7.6^†^ (0.050)**	1.5 (3)	0 (2)	−2 (3)	10.536* (0.005)	**6.509^#^ (0.049)**	**10.58‡ (0.001)**	4.071 (0.233)
ROCFT, Copy	1.5 (4.75)	.5 (9)	−0.5 (5.13)	3.820 (0.148)	46 (0.759)	24 (0.052)	33 (0.218)	1.5 (4)	0 (5)	−1.5 (4)	8.689* (0.013)	5.313 (0.112)	**9.813‡ (0.003)**	4.5 (0.192)
ROCFT, IR	1.25 (9.88)	1 (4)	−0.5 (6.75)	1.196 (0.550)	46 (0.762)	36.5 (0.307)	39.5 (0.426)	3 (6.13)	1.5 (5.5)	−1 (3.5)	4.637 (0.098)	23 (0.560)	11**‡** (0.047)	12 (0.109)

Note: AVLT = Auditory Verbal Learning Test; DR = Delayed Recall; IR = Immediate Recall; IQR = Interquartile range; KQ = Kruskal-Wallis; Mdn = Median; MoCA = Montreal Cognitive Assessment; MW = Mann-Whitney; NAIr = NeuroAIreh@b; PVF = Phonemic Verbal Fluency; PSI = Processing Speed Index; ROCFT = Rey-Osterrieth Complex Figure Test; SVF = Semantic Verbal Fluency; TG = Task Generator; TP = Toulouse-Piéron; TIR = Total Immediate Recall; TR = Total Result; WL = Waiting-List. *Significant group effect (Kruskal-Wallis, *p* < .05); ^#^NAIR≠TG (Mann-Whitney, *p* < .05); ^‡^NAIR≠WL (Mann-Whitney, *p* < .05); ^†^TG≠WL (Mann-Whitney, *p* < .05).

Furthermore, at three-months follow-up, a within-group analysis demonstrated that participants in the NAIr group showed statistically significant improvements in the MoCA (*W*_(8)_ = 26, *Z* = −2.032, *p* = .042, *r* = 0.72), TP-TR (*W*_(8)_ = 36, *Z* = −2.521, *p* = .012, *r* = 0.89), AVLT-TIR (*W*_(8)_ = 32, *Z* = −1.960, *p* = .05, *r* = 0.69), and AVLT-DR (*W*_(8)_ = 28, *Z* = 2.388, *p* = .017, *r* = 0.84), and maintained their performance in the remaining primary outcome measures (all *p values* ≥ 0.05). On the other hand, participants in the TG group only revealed significant gains in the MoCA (*W*_(7)_ = 25.50 , *Z* = −1.980; *p* = .048, *r* = . 75), and no other significant within-group changes were found (all *p values* ≥ 0.05), suggesting maintenance of performance in the other primary outcome measures. Conversely, the WL group demonstrated statistically significant declines in the MoCA (*W*_(7)_ = 21, *Z* = −2.214; *p* = .027, *r* = 0.84), PSI (*W*_(7)_ = 0, *Z* = −2.214; *p* = .027, *r* = 0.84), TP-TR (*W*_(7)_ =1.5, *Z* = −2.117; *p* = .034, *r* = 0.80), AVLT-TIR (*W*_(7)_ =0, *Z* = −2.375; *p* = .018, *r* = 0.90), and ROCFT-C (*W*_(7)_ = 0, *Z* = −2.207, *p* = .027, *r* = 0.83).

At follow-up, a Kruskal-Wallis test demonstrated that participants’ performance on several primary outcome measures was significantly affected by the type of treatment condition. Specifically, there were significant between-group differences in the MoCA (*χ2*_(2)_ = 11.076, *p* = .004), PSI (*χ2*_(2)_ = 9.659, *p* = .008), TP-TR (*χ2*_(2)_ = 11.235, *p* = .004), AVLT-TIR (*χ2*_(2)_ = 14.080, *p* = .001), AVLT-DR (*χ2*_(2)_ = 10.536, *p* = .005), and the ROCFT-C (*χ2*_(2)_ = 8.689, *p* = .013). The post hoc comparisons revealed that the participants in both the NAIr and the TG groups scored significantly higher than the WL group in the MoCA (NAIr vs. WL: *U* = 10.455, *Z* = −3.129, *p* = .002, *r* = 1.18; TG vs. WL: *U* = 8.929, *Z* = −2.587, *p* = .010, *r* = 0.98), and the PSI (NAIr vs. WL: *U* = 9.357, *Z* = −3.187, *p* = .003, *r* = 1.20; TG vs. WL: *U* = 8.024, *Z* = −2.342, *p* = .019, *r* = 0.89). Moreover, the NAIr group exhibited significantly better performances than the WL group in the TP-TR (NAIr vs. WL: *U* = 11.259, *Z* = −3.351, *p* = .001, *r* = 1.18), AVLT-TIR (NAIr vs. WL: *U* = 12.589, *Z* = −3.752, *p* < .001, *r* = 1.32), AVLT-DR (NAIr vs. WL: *U* = 10.58, *Z* = −3.201, *p* = .001, *r* = 1.13), and the ROCFT-C (NAIr vs. WL: *U* = 9.813, *Z* = −2.937, *p* = .003, *r* = 1.04). Finally, the NAIr group showed significantly higher improvements in the AVLT-DR compared to the TG group (NAIr vs. TG: *U* = 6.509, *Z* = −1.969, *p* = .049, *r* = 0.70).

### Secondary outcomes measures

The secondary outcome measures scores from the three groups in the different assessment moments are presented in Table 4. A within-group demonstrated that the NAIr group showed significant post-intervention improvements in the QOLIBRI (*W*_(10)_ = 47, *Z* = −1.989, *p* = .047, *r* = 0.63), the IAFAI – Global Functional Incapacity score (*W*_(10)_ = 0, *Z* = −2.666, *p* = .008, *r* = 0.84), and the MOT-Q (*W*_(10)_ = 44, *Z* = −2.561; *p* = .010, *r* = 0.81). As to the TG group, participants presented significant post-intervention improvements in the SMCS (*W*_(10)_ = 0, *Z* = −2.375, *p* = .018, *r* = 0.75) and the QOLIBRI (*W*_(10)_ = 28, *Z* = −2.371, *p* = .18, *r* = 0.75). On the other hand, the WL group only presented significant post-intervention declines in the IAFAI – Global Functional Incapacity (*W*_(10)_ = 15, *Z* = −2.023; *p* = .043, *r* = 0.64). The Kruskal-Wallis test revealed that some of the participants’ self-reported outcomes were significantly affected by the type of treatment condition (cf. Table 5). Specifically, there were significant post-intervention differences in the SMCS (*χ2*_(2)_ = 10.319, *p* = .006), QOLIBRI (*χ2*_(2)_ = 8.975, *p* = .011), IAFAI – Global Functional Incapacity (*χ2*_(2)_ = 16.452, *p* < 0.001), and the MOT-Q (*χ2*_(2)_ = 11.043, *p* = .004). Post hoc comparisons indicated that following the intervention, the TG group reported significantly greater improvements in self-reported memory complaints, as measured by SMCS, than both the NAIr and WL groups (NAIr vs. TG: *U* = −7.9, *Z* = −2.029, *p* = .042, *r* = 0.064; TG vs. WL: *U* = −12.35, *Z* = −3.171, *p* = .002, *r* = 1.00). However, the NAIr group presented significantly greater post-intervention improvements in functional abilities measured by the IAFAI – Global Functioning Incapacity than both the TG and the WL groups (NAIr vs. TG: *U* = −8.1, *Z* = −2.146 *p* = .032, *r* = 0.068; NAIr vs. WL: *U* = −15.3, *Z* = −4.054, *p* < .001, *r* = 1.28). Also, compared to the WL group, participants in the NAIr group reported significantly higher motivation for rehabilitation assessed by the MOT-Q (NAIr vs. WL: *U* = 12.833, *Z* = −3.322, *p* = .001, *r* = 1.05), and quality of life measured by the QOLIBRI (NAIr vs. WL: *U* = 11.55, *Z* = −3.926, *p* = .003, *r* = 0.93). Finally, the usability assessment, conducted only in the NAIr group, yield a median score of 82.50 (IQR = 37.88), indicating excellent usability based on SUS norms (90–95 percentile, Grade A rating).

**Table 4:** Secondary outcomes comparisons at pre, post-intervention and follow-up.

Measures	NAIr			TG			WL		
	**Pre (*n* = 10) Mdn (IQR)**	**Post (*n* = 10) Mdn (IQR)**	**FU (*n* = 8) Mdn (IQR)**	**Pre (*n* = 10) Mdn (IQR)**	**Post (*n* = 10) Mdn (IQR)**	**FU (*n* = 7) Mdn (IQR)**	**Pre (*n* = 10) Mdn (IQR)**	**Post (*n* = 10) Mdn (IQR)**	**FU (*n* = 7) Mdn (IQR)**
SMCS	5.5 (8.25)	4.5 (7.25)	6 (7.25)	7.50 (9)	6.00 (5)	5 (6)	6.00 (4.5)	9.00 (6)	10 (8)
*p* value*^w^*_(T0-T1)_	.857			.018*			.088		
*p* value*^w^*_(T0-T2)_	.102			.216			.066		
HADS	11.5 (9.75)	12 (15.25)	8 (18)	14 (14.75)	11.5 (10)	8 (8)	14.5 (11)	14 (9.5)	18 (17)
*p* value*^W^*_(T0-T1)_	.553			.261			.634		
*p* value*^W^*_(T0-T2)_	.779			.527			.172		
QOLIBRI	56.42 (22.85)	71.29 (23.32)	75.67 (27.71)	51.69 (31.93)	59.8 (30.4)	59.45 (30.41)	55.74 (13.61)	53.38 (12.84)	51.35 (15.89)
*p* value*^W^*_(T0-T1)_	.047*			.018*			.326		
*p* value*^W^*_(T0-T2)_	.091			.273			.144		
IAFAI	25.36 (23.74)	19.41 (18.59)	15.08 (15.55)	42.36 (29.8)	42.36 (34.7)	42.22 (9.6)	31.73 (28.93)	40.46 (29.71)	40 (25)
*p* value*^W^*_(T0-T1)_	.008*			.285			.043*		
*p* value*^W^*_(T0-T2)_	.018*			.527			.109		
MOT-Q	39 (13.25)	44.50 (13.5)	39 (19)	42.50 (18.75)	40 (17.5)	42 (15)	30.5 (19)	21 (13)	22 (24.75)
*p* value*^W^*_(T0-T1)_	.010*			.498			.078		
*p* value*^W^*_(T0-T2)_	.400			.248			.248		

Note: HADS = Hospital Anxiety and Depression Scale; IAFAI = Adults and Older Adults Functional Assessment Scale; MOT-*Q* = Motivation for Traumatic Brain Injury Rehabilitation QuestionNAIre; QOLIBRI = Quality of Life after Brain Injury; SMCS = Subjective Memory Complaints Scale; *p* value*^W^* = within-group changes (by time of assessment): *p < .05; **p < .01.

**Table 5:** Post hoc comparisons across groups considering the secondary outcome measures.

Measures	NAIr	TG	WL	KW (T1-T0)	MW NAIr vs. TG	MW NAIr vs. WL	MW TG vs. WL	NAIr	TG	WL	KW (T2-T0)	MW NAIr vs. TG	MW NAIr vs. WL	MW TG vs. WL
	**Mdn T1-T0 (IQR)**	**Mdn T1-T0 (IQR)**	**Mdn T1-T0 (IQR)**	***χ2 (p value)***	***U* (*p* value)**	***U* (*p* value)**	***U* (*p* value)**	**Mdn T2-T0 (IQR)**	**Mdn T2-T0 (IQR)**	**Mdn T2-T0 (IQR)**	***χ2 (p value)***	***U* (*p* value)**	***U* (*p* value)**	***U* (*p* value)**
SMCS	.5 (3.5)	−2 (5.5)	1 (2.25)	10.319* (0.006)	**7.9^#^ (0.042)**	7.9 (0.253)	**−12.35^†^ (0.002)**	−2 (−3.5)	−2 (3)	2 (3)	6.853* (0.032)	.527 (0.874)	**−7.402^‡^ (0.025)**	**−7.929^†^ (0.020)**
HADS	2 (10.75)	−1.5 (3.75)	−0.5 (6.75)	1.655 (0.437)	35 (0.255)	37 (0.325)	44 (0.648)	−1.5 (7.5)	−1 (7)	2 (5)	1.789 (0.409)	24.5 (0.685)	21.5 (0.448)	13.5 (0.158)
QOLIBRI	6.76 (9.8)	4.06 (6.68)	−1.69 (4.45)	8.957* (0.011)	3.9 (0.321)	**11.55^‡^ (0.003)**	7.65 (0.051)	3.38 (25)	0 (14.86)	0 (3.38)	6.202* (0.045)	**7.071^#^ (0.031)**	**7.071^‡^ (0.031)**	.000 (1)
IAFAI	−5.44 (8.05)	0 (1.59)	1.11 (3.53)	16.452* (<0.001)	**−8.1^#^ (0.032)**	**−15.3^‡^ (< 0.001)**	−7.2 (0.056)	−7.32 (11.06)	0 (12.76)	0 (6.38)	10.664* (0.005)	−3.304 (0.309)	**−10.446^‡^ (0.001)**	**−7.143^†^ (0.033)**
MOT-Q	5 (5.75)	1 (4.5)	0 (8)	11.043* (0.004)	5.850 (0.120)	**12.833^‡^ (0.001)**	6.983 (0.071)	3 (12.5)	1 (7)	0 (4)	.519 (0.772)	28.5 (0.750)	21.5 (0.513)	17.5 (0.612)

Note: HADS = Hospital Anxiety and Depression Scale; IAFAI = Adults and Older Adults Functional Assessment Scale; IQR = Interquartile range; KQ = Kruskal-Wallis; Mdn = Median; MW = Mann-Whitney; MOT-*Q* = Motivation for Traumatic Brain Injury Rehabilitation QuestionNAIre; NAIr = NAIr; TG = Task Generator; QOLIBRI = Quality of Life after Brain Injury; WL = Waiting-List. *Significant group effect (Kruskal-Wallis, *p* < .05); ^#^NAIR≠TG (Mann-Whitney, *p* < .05); ^‡^NAIR≠WL (Mann-Whitney, *p* < .05); SMCS = Subjective Memory Complaints Scale; ^†^TG≠WL (Mann-Whitney, *p* < .05).

At three-months follow-up, the within-group analysis demonstrated that participants in the NAIr group exhibited statistically significant improvements in the IAFAI – Global Functional Incapacity score (*W*_(8)_ = 0, *Z* = −2.366, *p* = .018, *r* = 0.84), and maintained their performance in the remaining secondary outcome measures (all *p values* ≥ 0.05). In contrast, neither the TG nor the WL group exhibited significant changes in self-reported outcomes at follow-up (all *p values* ≥ 0.05). This suggests that their initial self-reports remained stable over time (cf. Table 4). The Kruskal-Wallis test revealed that some secondary outcome measures were significantly affected by the type of treatment condition, namely the SMCS (*χ2*_(2)_ = 6.853, *p* = .032), QOLIBRI (*χ2*_(2)_ = 6.202, *p* = .045), and the IAFAI – Global Functional Incapacity (*χ2*_(2)_ = 10.664, *p* = .005) (cf. Table 5). Post hoc tests showed that participants in the WL group presented more severe self-reported subjective memory complaints measured by the SMCS (NAIr vs. WL: *U* = −7.402, *Z* = −2.237, *p* = .025, *r* = 0.84; TG x WL: *U* = −7.929, *Z* = −2.320, *p* = .020, *r* = 0.88), and greater functional disability assessed by the IAFAI – Global Functional Incapacity (NAIr vs. WL: *U* = −10.446, *Z* = −3.219, *p* = .001, *r* = 1.22; TG vs. WL: *U* = −7.143, *Z* = −2.131, *p* = .033, *r* = 0.80) when compared to participants previously enrolled in adaptive CT interventions. On the other hand, the NAIR group improved significantly more than both the TG and the WL groups in self-reported quality of life after brain injury, as assessed by the QOLIBRI (NAIr vs. TG: *U* = 7.071, *Z* = −2.157, *p* = .031, *r* = 0.76; NAIr vs. WL: *U* = 7.071, *Z* = −2.157, *p* = .031, *r* = 0.76).

## Discussion

In this study, we intended to compare and assess the efficacy of adaptive CT interventions delivered using the NAIr and the TG in chronic stroke survivors-related outcomes in relation to a passive control group. Hence, prior to the intervention process, all stroke survivors underwent a pre-neuropsychological assessment at baseline to establish their current level of abilities. Subsequently, chronic stroke survivors in the EGs were enrolled in 12 biweekly 30-minute sessions of adaptive CT. In contrast, survivors in the passive control group did not receive any non-pharmacological intervention during the study. Post-intervention and three-month follow-up assessments were conducted on all stroke survivors to evaluate the immediate and medium/long-term effects of both adaptive CT modalities. The following sections will present our results regarding the effects of adaptive CT in stroke survivors’ primary and secondary outcome measures.

### Feasibility outcomes

The high rates of completion at post-intervention and three-month follow-up, as well as participants’ adherence to the training regimen, demonstrate the feasibility of both NAIr and TG in community-dwelling chronic stroke survivors.

### Primary outcome measures

Overall, at post-intervention, stroke survivors in the NAIr group presented different within-group significant improvements in several cognitive outcome measures, namely global cognition, sustained and selective attention, visuoconstructional abilities and executive functions. In contrast, the TG group showed significant within-group improvements in sustained and selective attention, and executive functions (i.e., phonemic verbal fluency) measures. On the contrary, participants in the passive control group exhibited a significant decline in their performance on both semantic verbal fluency and episodic verbal memory (i.e., delayed recall). Between-group comparisons revealed significantly greater cognitive improvements in participants assigned to adaptive CT conditions. Indeed, compared to the WL group, those in the NAIr and TG groups demonstrated a superior performance in measures of global cognition, processing speed, sustained and selective attention, verbal episodic memory (immediate recall and delayed recall), and executive functions (i.e., phonemic verbal fluency). Notably, the WL control group demonstrated a significant decline in processing speed compared to the NAIr group.

At three-month follow-up, the NAIr group demonstrated further significant improvements in global cognition, sustained and selective attention measures, and new improvements emerging at this stage in episodic verbal memory (immediate recall and delayed recall). On the other hand, the TG group only presented significant improvements in global cognitive functioning, while the WL group exhibited a significant decline in the following cognitive domains: global cognition, processing speed, sustained and selective attention, episodic verbal memory (i.e., immediate recall), visuoconstructional abilities and executive functions. Moreover, between-group comparisons revealed that participants allocated to the adaptive CT interventions continued to outperform participants in the WL group in measures of global cognition and processing speed. It was also found that the NAIr group outperformed the WL group in sustained and selective attention, episodic memory (immediate recall and delayed recall), visuoconstructional abilities and executive function measures. Finally, the NAIr group also showed significantly greater improvements in delayed recall relative to the TG group.

There are some discrepancies between our findings and those of Faria et al. (2020). First, we can assume that our results are partially aligned with Faria et al. (2020) when considering the broader effects of the ICT-based intervention on stroke survivors' cognitive functioning. Indeed, similarly to what was verified by Faria et al. (2020), adaptive CT delivered via the NAIr platform led to more significant post-intervention improvements in stroke survivors' cognitive functioning, including global cognition, attention, visuoconstructional abilities and executive functions, relative to the paper-and-pencil CT intervention, which accounted for less improvements on a cognitive level. Second, in contrast to Faria et al. (2020), who reported a between-group difference in global cognition at post-intervention, in our study, we concluded that survivors in either adaptive CT method – the NAIr or the TG – demonstrated significant improvements in many cognitive domains than survivors in the control group receiving no intervention. Third, another contrasting finding among our study and Faria et al. (2020) is related to the interventions' effects at follow-up. While the TG group in Faria et al. (2020) retained verbal memory improvements and demonstrated further significant improvements in sustained attention, processing speed, and language at a two-month follow-up, our study revealed a greater number of within-group improvements for the NAIr group compared to the TG. Furthermore, it identified a potential advantage for the NAIr intervention in improving stroke survivors' verbal memory, specifically delayed recall, at three-month follow-up. It is important to note that Faria et al. (2020) attributed the lack of significant improvements in their VR group at the two-month follow-up to a high dropout rate (64.29 %) compared to the TG (0 %). Conversely, our study maintained a similar dropout rate in both the NAIr (20 %) and the TG group (30 %). This latter factor, i.e., dropout rate, along with the other methodological differences (e.g., ICT-based intervention administered, primary outcome measures used, absence of a passive control group), may explain the observed discrepancies between both studies.

### Secondary outcome measures

In addition to cognitive functioning, we measured the adaptive CT intervention’s impact on several noncognitive outcome measures. These included self-reported subjective memory complaints, anxiety and depressive symptomatology, quality of life, functional abilities, and motivation for rehabilitation. Furthermore, we performed a usability evaluation specifically for stroke survivors in the NAIr group.

Immediately after the intervention, the NAIr group showed significant within-group improvements in self-perceived quality of life after brain injury, functional abilities and motivation for rehabilitation, while the TG group reported similar quality of life benefits and fewer subjective memory complaints. Conversely, the WL group reported significantly greater functional disability at post-intervention. Moreover, a between-group comparison considering participants’ self-reports from baseline to post-intervention suggested that participants in the NAIr group reported significantly less functional disability compared to both the TG and WL groups, as well as greater motivation for rehabilitation than survivors in the WL group. Interestingly, the TG group reported significantly fewer subjective memory complaints than both the NAIr and the WL groups.

At three-month follow-up, a within-group analysis showed that only participants in the NAIr group reported further improvement in their self-perceived functional abilities. There were no significant changes in any of the secondary outcome measures for participants in the other groups. Comparing self-reported outcomes from baseline to follow-up across groups, we found that the WL group experienced a significant increase in both subjective memory complaints and functional disability when compared to participants enrolled in both intervention conditions. Also, a between-group comparison demonstrated that the NAIr group reported a significantly higher quality of life relative to the WL and the TG groups.

Overall, our initial findings suggest that the cognitive benefits experienced by stroke survivors following adaptive CT through the NAIr platform appeared to generalize to a wider range of noncognitive domains (i.e., quality of life, functional abilities and motivation), comparatively to the TG (i.e., quality of life, subjective memory complaints) at post-intervention. In addition, the NAIr group reported significantly less functional disability than the TG group immediately after the intervention, having maintained these results at three-month follow-up. Concerning the latter finding, the research on the effects of ICT-based CT interventions on patients’ functional abilities remains inconclusive (Câmara, Lan-Ju et al., 2023; Paulino et al., 2022; Pinto et al., 2024; Zhou et al., 2022). For instance, despite Zhou et al. (2022) identified cognitive gains in stroke survivors following CCT, these gains did not translate into significant changes in ADLs. In contrast, in a previous pilot study with the prototype version of the NAIr platform, stroke survivors’ global cognitive improvements at post-intervention (assessed by the MoCA) generalized to functional abilities (measured by the IAFAI) (Câmara, Paulino et al., 2022). It is assumed that enhancing the ecological validity of neuropsychological interventions can facilitate the generalization of training effects beyond the trained tasks (Câmara, Ferreira et al., 2022; Faria et al., 2016; Oliveira et al., 2020; Sohlberg & Turkstra, 2011). Hence, driven by technological innovations in the field of neuropsychological rehabilitation, a growing body of research focuses on the development and clinical validation of more ecologically valid intervention methods (e.g., VR environments incorporating CT tasks resembling IADLs such as meal preparation, navigation in a virtual city) with the promise of proving a more cost-effective training experience) (Faria et al., 2024; Oliveira et al., 2020; Parsons, 2016; Riva et al., 2020). A recent systematic review conducted by Pinto et al. (2024) identified that, despite many class I studies employing what they reported to be more ecologically valid intervention methodologies (e.g., VR-based interventions simulating ADLs), many studies did not assess the impact of the intervention on patients’ ADL performance or participation outcomes. Additionally, some studies reported the lack of significant differences between the intervention and the control groups in measures of social participation and real-life tasks, highlighting the need for a clear definition of ecological validity in neuropsychological interventions, as well as a standardized checklist to operationalize this construct when designing new interventions or evaluating already developed interventions.

Moreover, we hypothesize that improvements in functional abilities in the NAIr group might have contributed to significantly greater gains in stroke survivors’ quality of life at three-month follow-up. These findings are consistent with those of Faria et al. (2020), who found that only the VR-based intervention significantly reduced self-perceived cognitive deficits in different aspects of stroke survivors’ everyday lives, including everyday life skills, family and life, mood, and sense of self. Despite the authors have used a different outcome measure to assess the impact of PSCI in patients’ everyday life – the Patient-reported evaluation of cognitive state (PRECiS) – the items of this self-report measure exhibit similarities to those incorporated in the QOLIBRI, which are organized in the following core domains: cognition, self, daily life and autonomy, social relationships, emotions and physical problems.

Finally, considering the usability assessment of the NAIr platform, stroke survivors reported an excellent level of usability, representing a significant improvement over the prototype version of the NAIr platform, which received acceptable usability ratings in a previous pilot study (41‑59 percentile, C grade) (Paulino, Câmara, Branco et al., 2022). These findings are in line with prior research from our laboratory, corroborating the user-friendliness of other ICT-based CT tools designed for stroke patients (Faria et al., 2020; Vourvopoulos et al., 2014).

Notwithstanding, this study is not without limitations, thus the findings must be interpreted with caution. As outlined in the methods section, this is an ongoing investigation with a currently small sample size. To address this limitation and enhance the generalization of our findings to the broader community of chronic stroke survivors, we plan to expand our sample size in the upcoming months. This will mitigate the potential for Type II errors (i.e., failing to detect a true treatment effect) due to insufficient statistical power associated with nonparametric tests, which are often more conservative (Faria, 2020). Some additional limitations can be pointed out. For instance, this study employed a non-randomized WL control group consisting of participants who were not able to enroll in the NAIr or TG interventions. While this approach was necessary due to practical constraints during sample recruitment, it introduces additional bias (e.g., nocebo effect) (Bagarić et al., 2022; Locher et al., 2019). Specifically, given the control group’s awareness of the ongoing interventions, their performance on cognitive and noncognitive outcome measures may have been negatively influenced. This could be due to participants anticipating a decline in their cognitive abilities, which influenced their performance during the neuropsychological assessments. In addition, it is important to note that despite having included a passive control group, the inclusion of an active control group, such as a non-adaptive CT approach, would have strengthened the study design by enabling a direct comparison of the efficacy of adaptive versus non-adaptive CT methods. Moreover, its important to note that only participants were blinded to the experimental conditions. Another potential source of bias in the current study is that the psychologist responsible for both the neuropsychological assessments and the training sessions was aware of part participants’ allocation. Furthermore, in this pilot study we did not conduct an intention-to-treat analysis, as we have only analyzed data from participants who completed baseline, post-intervention and follow-up neuropsychological assessments. This approach may hinder the generalizability of our results, since it does not account for data from participants who dropped out of the study.

Another important aspect to highlight is the chronicity of the deficits presented by community-dwelling stroke survivors; scientific literature emphasizes that restorative intervention techniques like CT tend to be more effective when interventions start early in the stroke rehabilitation continuum, enabling therapists to take advantage of the brain’s spontaneous recovery processes (Sohlberg & Mateer, 2001; Sohlberg & Turkstra, 2011). However, recruiting patients during the subacute phase proved infeasible due to some practical constraints. One primary concern was the demanding nature of existing rehabilitation programs. Patients with moderate to severe strokes typically undergo intensive inpatient rehabilitation in the subacute phase, encompassing occupational therapy, physiotherapy, and speech and language therapy. Introducing an additional intervention like CT during this period could exacerbate patients’ fatigue, which is a prevalent and disabling post-stroke symptom. Increased fatigue might hinder patient participation in rehabilitation and ultimately compromise rehabilitation outcomes (Crosby et al., 2012; Lanctôn et al., 2020; Miller, 2013). Therefore, it is critical to find the optimal balance between maximizing therapeutic benefit and minimizing patient burden. Second, if CT had been incorporated into the existing rehabilitation regimen, isolating its specific effects would be challenging due to the multifaceted nature of the interventions and the potential for spontaneous recovery.

Consequently, focusing on chronic stroke survivors minimizes the confounding variables previously mentioned. Moreover, even though restorative interventions like CT may be less effective in the chronic stage, there is evidence suggesting that chronic stroke survivors can still benefit from CT on a cognitive level (Amiri et al., 2021; Cicerone et al., 2019; Faria et al., 2020; Gil-Pagés et al., 2022; Veisi-Pirkoohi et al., 2020). Indeed, CT plays a crucial role in training and maintaining cognitive function, and structured, hierarchically driven stimulation can help prevent pathological aging trajectories, potentially mitigating the development of vascular cognitive impairment or vascular dementia in later life.

## Conclusions

In summary, we found support for the feasibility of both the NAIr and the TG interventions. Moreover, preliminary efficacy findings from this three-month longitudinal RCT indicate that adaptive CT methods contribute to immediate and medium/long-term positive effects in cognitive and noncognitive domains of community-dwelling stroke survivors. Furthermore, these results suggest that the NAIr intervention, incorporating ADL-oriented training tasks, was the only adaptive CT approach to promote the generalization of cognitive improvements to functional abilities and quality of life at post-intervention and three-month follow-up, respectively. A larger scale RCT is currently ongoing in two other Portuguese healthcare institutions to further validate these preliminary findings in a more representative sample of community-dwelling stroke survivors.

## Funding

This work was supported by the 10.13039/501100001871Fundação para a Ciência e a Tecnologia (FCT) under the BRaNT project (PTDC/CCICOM/30990/2017), PhD grants (SFRH/BD/145919/2019) awarded to JC and (SFRH/BD/147390/2019) to TP. This work was further supported by research units NOVA LINCS reference UIDB/04516/2020 (https://doi.org/10.54499/UIDB/04516/2020) and reference UIDP/04516/2020 (https://doi.org/10.54499/UIDP/04516/2020) NOVA LINCS reference: BRaNT project PTDC/CCI-COM/31046/2017.

## Declaration of competing interest

The authors declare the following financial interests/personal relationships which may be considered as potential competing interests: Joana Câmara, Teresa Paulino e Eduardo Fermé report financial support was provided by Fundação para a Ciência e Tecnologia (FCT IP). The remaining authors declare that they don’t have any known competing financial interests or personal relationships that could have appeared to influence the work reported in this paper.

## Appendix Supplementary materials

Image, application 1

## Footnotes

1 Training manual available at: https://neurorehabilitation.github.io/TaskGenerator/.
